# A Review of Clinical Outcomes of CAR T-Cell Therapies for B-Acute Lymphoblastic Leukemia

**DOI:** 10.3390/ijms22042150

**Published:** 2021-02-21

**Authors:** Massimo Martino, Caterina Alati, Filippo Antonio Canale, Gerardo Musuraca, Giovanni Martinelli, Claudio Cerchione

**Affiliations:** 1 Stem Cell Transplant and Cellular Therapies Unit, Hemato-Oncology and Radiotherapy Department, “Bianchi-Melacrino-Morelli” Hospital, 89124 Reggio Calabria, Italy; filcan87@gmail.com; 2 Hematology Unit, Hemato-Oncology and Radiotherapy Department, “Bianchi-Melacrino-Morelli” Hospital, 89124 Reggio Calabria, Italy; caterina.alati@gmail.com; 3 Hematology Unit, IRCCS Istituto Scientifico Romagnolo per lo Studio dei Tumori (IRST) “Dino Amadori”, 47014 Meldola, Italy; gerardo.musuraca@irst.emr.it (G.M.); giovanni.martinelli@irst.emr.it (G.M.)

**Keywords:** B-acute lymphoblastic leukemia, chimeric antigen receptor (CAR-T), relapsed and refractory ALL, CD19-targeted CAR-modified T-cell

## Abstract

Introduction: Treatment of relapsed and refractory (R/R) B acute lymphoblastic leukemia (B-ALL) represents an unmet medical need in children and adults. Adoptive T cells engineered to express a chimeric antigen receptor (CAR-T) is emerging as an effective technique for treating these patients. Areas covered: Efficacy and safety of CAR-T therapy in R/R B-ALL patients. Expert opinion: CD19 CAR-T infusion induce high CR rates in patients with poor prognosis and few therapeutic options, while real-life data demonstrate similar results with an interestingly lower incidence of grade 3/4 toxicity. Nevertheless, despite impressive in-depth responses, more than half of patients will experience a relapse. Therefore, rather than using CAR-T cell therapy as a stand-alone option, consolidation with allogeneic stem-cell transplant (Allo-SCT) after CAR-T treatment might increase long-term outcome. Moreover, CD19 is one target, but several other targets are being examined, such as CD20 and CD22 and dual-targeting CARs or combination therapy. Another issue is the time consuming process of CAR-T engineering. New platforms have shortened the CAR-T cell manufacturing process, and studies are underway to evaluate the effectiveness. Another way to mitigate waiting is the development of allogeneic “off the shelf” therapy. In conclusion, CD19-targeted CAR-modified T-cell therapy has shown unprecedented results in patients without curative options. Future work focusing on target identification, toxicity management and reducing manufacturing time will broaden the clinical applicability and bring this exciting therapy to more patients, with longer-term remissions without additional Allo-SCT.

## 1. Introduction

Acute lymphoblastic leukemia (ALL) is a rare malignancy with 16 years of median age at diagnosis [1,2]. The first classification of ALL was the French American British (FAB) that used morphological criteria, identifying three subtypes (L1, L2, and L3) [3]. Twenty years later, the World Health Organization published a classification that considers both morphology and cytogenetic characteristics and identified three types of ALL: B lymphoblastic, T lymphoblastic, and Burkitt-cell Leukemia [4]. Later, Burkitt-cell Leukemia was not considered a separate entity from Burkitt Lymphoma, classifying B-lymphoblastic leukemia into two subtypes: B-ALL with genetic abnormalities, and B-ALL not otherwise specified. B-ALL with genetic abnormalities is further delineated based on the specific chromosomal rearrangement present [5].

In the modern classification, two new categories were added to the genetic abnormalities, and the hypodiploid was redefined as either hypodiploid or low hypodiploid with TP53 mutations [6]. 

ALL is the second most common acute leukemia in adults, with an incidence of over 6500 cases per year in the United States. In adults, 75% of cases develop from precursors of the B-cell lineage, with the remainder of cases consisting of malignant T-cell precursors. Despite advances in management, the backbone of treatment remains multi-agent chemotherapy with vincristine, corticosteroids and an anthracycline with allogeneic stem cell transplantation (Allo-SCT) for eligible patients [7]. Although rates of complete remission (CR) are high, long-term disease-free survival (DFS) can be obtained in only about 40% of adult patients [8,9,10]. Fielding and colleagues published the outcome of 609 adults with recurring ALL, where the overall survival (OS) after relapse was globally 7% [11]. Factor predicting a better outcome after salvage therapy was the young age, with OS of 12% and 3% in patients younger than 20 years and older than 50 years, respectively. In another study, Tavernier and colleagues showed that the prognosis of recurrent ALL in adult patients is globally dismal [12]. 

It is evident that treatment of relapsed/refractory (R/R) B-ALL disease represents an unmet medical need, and prospective studies involving novel therapeutic agents should be improved. Agents such as monoclonal antibodies (anti-CD20), anti-CD19 bispecific T-cell engager, and anti-CD22 antibody-drug conjugate have shown unexpected results in both upfront and R/R setting and continue to change the treatment paradigm for ALL [13,14,15]. In a phase 3 trial, inotuzumab ozogamicin (an anti-CD22 antibody conjugated to calicheamicin) demonstrated a significantly higher remission rate, and a better progression-free survival (PFS) compared with standard intensive chemotherapy in adults with R/R B-cell ALL. Moreover, more patients achieved MRD-negativity and proceeded to Allo-SCT (13). In another randomized phase 3 trial involving adults with Ph-negative R/R B-cell precursor ALL, blinatumomab (a bispecific monoclonal antibody construct that enables CD3-positive T cells to recognize and eliminate CD19-positive ALL blasts), resulted in significantly longer overall survival (OS) than standard chemotherapy, and the risk of death was 29% lower in the blinatumomab group than in the chemotherapy group (14). Recent data from multiple studies positioned chimeric antigen receptor-modified T (CAR-T) therapy with a significant edge over these novel agents due to better outcomes in R/R ALL. While CAR-T therapies targeting the CD19 antigen have produced durable remissions, potentially severe toxicities may limit their use. Moreover, less discussed is what can happen during the period in which patients wait for CAR-T cells to be processed and engineered. This bridging period (2–4 weeks) can leave R/R B-ALL patients extremely vulnerable and there is always a risk of progression during a time of no treatment. In this paper, we focus on current evidence in the literature for the efficacy and safety of CAR-T therapy in B-ALL. 

## 2. CAR-T Constructs

CARs are genetically engineered T cells that express the antigen-binding domain of an immunoglobulin linked via transmembrane domains to the intracellular T-cell receptor signaling parts [16]. This construct allows the T cells to recognize unprocessed antigens and be activated in a major histocompatibility complex (MHC)-independent way [17]. Basic CARs structure include an intracellular T-cell activation domain, an extracellular hinge region, a transmembrane domain, and an extracellular antigen-recognition moiety that derives from an antibody (single-chain variable fragment (scFv)) [18]. The hinge, or spacer element, is conceived to optimize the accessibility of the epitope. Second-generation CAR-T cells encode for a co-stimulatory domain, such as CD2822, or members of the tumor necrosis factor receptor family such as CD137 (4-1BB) [19]. This co-stimulation domain provides better cytokine production and proliferation and enhances CAR-T cells’ persistence [20,21]. Third-generation CARs include a second co-stimulatory domain using the co-stimulatory mentioned above domains or others as CD27, ICOS, or CD134 (OX40) [22]. CAR T-cell therapy involves collecting T cells, introducing the CAR construct, and then, after lymphodepletion, the infusion of the modified T cells in the patient. The main characteristics and targets of the CAR-T cells are summarized on Table 1.

**Figure 1:**
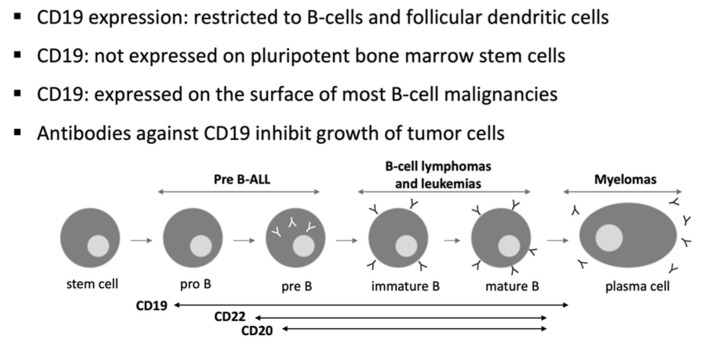
CD19: target for immunotherapy against B-cell ALL.

CD19 is a target for immunotherapy against B-cell ALL due to its near-universal expression on B-lymphoblasts [23,24] (Figure 1). The idea of adoptive immunotherapy using lymphocytes to attack leukemia was born in the early 1990s. After cloning the T cell antigen receptor’s zeta-chain, the first chimeric antigen receptor was conceived by Eshhar et al. [25]. Many molecular and configurational modifications have been attempted with this product to optimize its antitumor efficacy [26]. Many North American groups, such as Memorial Sloan Kettering Cancer Center (MSKCC), University of Pennsylvania (UPenn), Children’s Hospital of Philadelphia (CHOP), Fred Hutchinson Cancer Research Center (FHCRC), and the National Cancer Institute (NCI), have developed CAR-T products and started clinical trials with anti-CD19 therapies for B-cell malignancies [27]. In 2012, UPenn was the first to create a research alliance with a pharmaceutical company to develop CAR-T cells for commercialization after its initial clinical success [27]. CTL019, later known as tisagenlecleucel (Tisacel), was the first CAR-T treatment approved by the US Food and Drug Administration (FDA). The initial results of CTL019 in ALL were published in 2013 [28]. Since then, many trials are ongoing with various CAR-T products for different indications and with promising results. 

## 3. Anti-CD19 CAR T-Cell Studies

The main results are summarized on Table 2 and Table 3. Park and colleagues published the results of a phase I trial in which 53 R/R B-ALL adult patients, with and a median age of 44 years (range 23–74), received an infusion of CAR-T expressing the 19–28z CAR [29]. The overall CR rate was 83%, with a negative MRD in 66.6% of evaluable patients (*n* = 48). With a median follow-up of 29 months, the median EFS and OS were 6.1 and 12.9 months, respectively. The authors showed that in vivo peak CAR T-cell expansion was the best predictor of response and toxic effects and that patients with a low disease burden had a significantly longer EFS and OS. 

The group of FHCRC reported a high rate of CR with negative MRD (93%) in adult R/R B-ALL patients who received an infusion of CD19 CAR T cells with a 4-1BB co-stimulatory [30]. The same authors published the long-term follow-up of these patients and analyzed factors associated with durable EFS [31]. Forty-five (85%) of 53 patients evaluable for response achieved a CR with negative MRD. With a median follow-up of 30.9 months, EFS and OS were significantly better in the group who achieved a CR with negative MRD than those who did not (7.6 vs. 0.8 months; *p* < 0.0001 and 20.0 vs. 5.0 months; *p* = 0.014, respectively). In multivariate analysis, the factors associated with better EFS were: a lower LDH concentration, higher platelet count before lymphodepletion, and use of fludarabine in the lymphodepletion regimen. Despite the high CR rate with a negative MRD, there was a significant risk of relapse (49%). The occurrence of relapse with the negativity of CD19 in patients with a substantial CAR T-cell expansion and persistence was consistent with continued immune pressure, suggesting that strategies to minimize escape by antigen loss [32]. 40% of patients with a CR and a negative MRD after CAR T-cell infusion underwent allo-SCT. In this setting, the authors observed a significantly better EFS. The conclusions were that CD19 CAR T-cells were highly effective at inducing a CR with negative MRD, but relapse was frequent, especially in the absence of allo-SCT. 

A phase 1 trial evaluated CAR-T infusion in 21 young patients (aged 1–30 years) with R/R B-ALL or non-Hodgkin lymphoma, including eight who had previously undergone Allo-SCT [33]. CAR-T cells were engineered with a manufacturing process to express a CD19-CAR incorporating an anti-CD19 single-chain variable fragment plus CD3zeta and CD28 signaling domains. The maximum tolerated dose was 1 × 106 CD19-CAR T cells per kg. All toxicities were fully reversible, with the most severe grade 4 CRS in 3 of 21 patients. The most common non-hematological grade 3 adverse events were: neutropenic fever (43%); hypokalemia (43%); fever and neutropenia (38%); and CRS (14%). In B-ALL patients, the CR rate was 70%, with a negative MRD rate of 60%. Ten of 12 patients who became MRD-negative received an Allo-SCT, and all remain disease-free (median follow-up ten months). The authors concluded that CD19-CAR T-cell therapy could be an effective bridge to Allo-SCT in patients who achieved a CR, or, better, a MRD-negative status. 

A phase 1 trial of young patients with R/R B-ALL was performed using a uniform CD19 CAR expression product of defined CD4/CD8 composition, with limited effector differentiation [34]. The overall MRD negative remission rate was 89%, with 100% in the subset of patients who received fludarabine and cyclophosphamide lymphodepletion. Twenty-one percent of patients developed severe reversible immune effector cell-associated neurotoxicity syndrome (ICANS), with no deaths attributable to product toxicity. With a median follow-up of 9.6 months, the estimated 12-month EFS and OS were 50.8% and 69.5%, respectively. The loss of functional CAR T cells adversely affected the risk of CD19-leukemic relapse. This trial demonstrated that manufacturing a defined-composition CD19 CAR T cell is a strategy for having a CAR T with highly potent antitumor activity and a tolerable adverse effect profile.

Moreover, the study identified a subset of patients with a durable remission enhanced by ongoing CAR T-cell persistence. A risk factor for relapse with a disease CD19- was a short duration of B-cell aplasia that can be predicted by the quantity of CD19- expressing B cells (malignant and nonmalignant) in the bone marrow before CAR T-cell dosing. The authors observed that low quantities of CD19-B cells resulted in suboptimal expansion and persistence of CD19 CAR T cells, indicating a future challenge when this modality is applied among patients earlier in the course of their therapy. The conclusions were that approaches to augment CD19 CAR T-cell persistence independent of B-cell/leukemic cell burden should be warranted. It should be pursued via the provision of T cells transduced to express CD19 after CAR T-cell infusion. Moreover, the use of fludarabine and cyclophosphamide lymphodepletion should promote longer persistence of the CAR T cells.

Tisacel is a second-generation CAR-T cell constructed with activating (CD3ζ) and co-stimulatory signals (4-1BB). This activation then leads to T-cell proliferation, cytokines secretion, and cytolysis. Results from a single-center phase 1-2a study of CTL019 involving 60 children and young adults with R/R B-cell ALL conducted by CHOP and the UPenn showed a rate of CR of 93% [35]. CRS occurred in 88% of patients and was effectively managed with supportive measures and anti-cytokine therapy. Long-term disease control without additional treatment and with the persistence of Tisacel for up to 4 years has been observed [36].

The same group designed a nonrandomized Tisacel therapy study (ELIANA trial), using a global supply chain and 25 study sites in 11 countries across North America, Europe, Asia, and Australia [37]. Seventy-five children and young adults with R/R B-cell ALL (61% of whom had had a relapse after Allo) received an infusion of Tisacel and could be evaluated for efficacy. Within three months, the ORR was 81%, with all patients who had a response to treatment found to be MRD negative. EFS and OS rates were 73% and 90%, 50% and 76%, at 6 and 12 months, respectively. The persistence of Tisacel in the blood was observed for as long as 20 months. CRS occurred in 77% of patients, 48% of whom received tocilizumab. ICANS occurred in 40% of patients and were managed with supportive care. Tisacel persistence was observed more than one year after infusion in patients with treatment response. This finding indicated that patients could be effectively treated with Tisacel across a wide dose range without an apparent expansion and response effect. The authors concluded that Tisacel produced high remission rates and durable remissions without additional therapy. The latest analysis of the ELIANA trial results included four additional patients and another follow-up year [38]. Results were presented by Grupp et al. at the 2018 American Society of Hematology (ASH) Annual Meeting. ORR was 82%, and 62% of patients had a CR. 66% of patients who had a CR were still in remission at 18 months, and the ORR was 70% at 18 months post-infusion, with amedian OS not reached. 

At the 2019 American Society of Clinical Oncology Annual Meeting, Dr. Shah and colleagues presented the results of ZUMA-3, a single-arm Phase 1/2 study in adult patients with R/R B-ALL. They used KTE-X19 (Brexucabtagene Autoleucel), an investigational anti-CD19 single-chain variable fragment (scFv) coupled to the co-stimulatory signaling domain CD28 and the zeta chain of the T-cell receptor complex (CD3 zeta) [39]. By the end of Phase 1, 45 patients received KTE-X19 at one of three different doses levels (2 × 10^6^ cells/kg (*n* = 6), 1 × 10^6^ cells/kg (*n* = 23), or 0.5 × 10^6^ cells/kg (*n* = 16)). Of 41 patients who were evaluable for efficacy, 68% achieved CR or CR with incomplete hematological recovery (CRi), and 100% of responders had a negative MRD. Of the 23 patients treated with the dose level used in the ongoing Phase 2 study, 19 were evaluable for efficacy, and 16 patients achieved CR or CRi, and 12 patients were in ongoing response, with a median duration of remission of 12.9 months). Grade ≥3 CRS and ICANS occurred in 29% and 38% of all patients, respectively. Two patients experienced Grade 5 adverse events; 1 developed stroke in the context of CRS and ICANS, and one experienced multiorgan failure post-CRS. Among patients receiving 1 × 10^6^ cell/kg (*n* = 23), 26% experienced grade ≥3 CRS, and 43% experienced grade ≥3 ICANS. The authors implemented a revised AE management protocol in 9 patients treated with 1 × 10^6^ cell/kg of KTE-X19 during the study. In this revised protocol, corticosteroids were initiated at the onset of grade ≥2 ICANS, and tocilizumab was given to manage toxicities in the context of CRS. Of those patients, 2 had grade 3 CRS, and 1 had grade 3 neurologic events. There were no grade 4/5 events.

When Tisacel was used in the real-world setting, efficacy outcomes were similar, and safety was more favorable than the pivotal trial. At the 2020 ASH Annual Meeting, Schultz et al. reported real-world clinical outcomes using commercially available Tisacel for pediatric R/R B-ALL [40]. Retrospective data were collected from 15 institutions and included 185 patients infused with Tisacel, including 161 receiving standard-of-care CAR T cell products meeting manufacturing release criteria and 24 receiving CD19-CAR T cells manufactured and provided on the managed access program (*n* = 14) or with single-patient IND approval (*n* = 10). At the time of CAR T cell infusion, the median age was 12 years (range 0–26). Early responses at one month and OS and EFS at 6 and 12 months are comparable to reported ELIANA trial outcomes. The rate or CRS and ICANS was low. Comparative analysis of outcomes in patient cohorts with varying disease burden demonstrates decreasing CR, EFS, and OS in patients with high disease burden compared to patients with lower disease burden or no detectable disease at last evaluation before CAR infusion. Recently, Pasquini et al. published the largest set of safety and efficacy data for Tisacel in a real-world setting from a cellular therapy registry [41]. 410 patients had follow-up data reported (ALL, *n* = 255; NHL, *n* = 155). Among patients with ALL, the initial CR rate was 85.5% vs. 82.3% observed in the ELIANA trial. Twelve-month duration of response (DOR), EFS, and OS rates were 60.9%, 52.4%, and 77.2%, respectively vs. 67.4%, 57.2%, and 77.1% observed in the Eliana trial. Grade ≥3 CRS and ICANS were reported in 16.1% and 9.0% of patients, respectively, compared with 48.1% and 12.7% of the ELIANA study. Pivotal Tisacel trial did not include children <3 years of age; however, almost 6% of the ALL real-world cohort were age <3 years. Prior treatment with Allo-SCT was less frequent among patients in this study than the Eliana trial (28% vs. 61%). Primary refractory patients were more common in the registry than in the pivotal trial (15% vs. 8%). The real-world analysis used the grading scales ASTCT for CRS and ICANS for neurologic events. In contrast, the ELIANA trial used the Penn Grading Scale for CRS and MedDRA SQM neurologic events. The NCI and Penn grading systems included a four level scale of severity with some differences. ASTCT developed a common grading system for both CRS and neurotoxicity. In this scale, CRS grading is driven by hypotension and/or hypoxia and CRS grade is determined by the more severe event.

Recently, Anagnostou et al. published the results of a meta-analysis of published and unpublished clinical trials that reported data on the outcomes of 953 patients treated with anti-CD19 CAR T cells R/R B-ALL [42]. The meta-analysis showed that the rate of pooled CR was 80% (95% CI 75·5–84·8), 1-year PFS was 37% (28·1–47·0), and the activity of anti-CD19 CAR T-cell therapy was similar for adults and children. Analysis by anti-CD19 CAR T-cell construct type revealed that a higher proportion of patients treated with 4-1BB co-stimulated constructs had undetectable MRD than did patients treated with CD28 co-stimulated constructs. Additionally, compared with patients treated with allogeneic anti-CD19 CAR T cells, patients treated with autologous CAR T cells had improved CR and were more likely to have ICANS. Treatment with different anti-CD19 CAR T-cell constructs was not associated with significant differences in the proportion of patients with CRS or ICANS.

Zhang et al. published the result of a phase 1/2 study using anti-CD19 CAR T cells to treat patients with R/R B-ALL, including those with high-risk features such as BCR-ABL fusion gene, TP53 mutation (12 patients), extramedullary disease (EMD) (including central nervous system [CNS] leukemia), and those who relapsed after allo-SCT (16 patients) [43]. Kaplan-Meier analysis showed that OS and LFS at six months were much lower for patients carrying the TP53 mutation than the patients without a TP53 mutation (OS: 51.9% vs. 89.0%; *p* =0.0001; LFS: 42.4% vs. 82.6%; *p* = 0.0002). Patients with a previous transplantation history had a lower 1-year OS and LFS than the group without a previous transplant (OS: 30.5% vs. 79.2%; *p* = 0.009; LFS: 25.4% vs. 69.4%; *p* = 0.011). Of the 102 CR patients, 75 nonrandomly selected patients subsequently proceeded to allo-SCT. Kaplan-Meier analysis showed that 1-year OS and LFS for patients proceeded allo-SCT after CAR T-cell therapy were significantly better than those for patients receiving CAR T-cell therapy alone (OS: 79.1% vs. 32.0%; *p* = 0.0001; LFS: 76.9% vs. 11.6%; *p* = 0.0001). The authors concluded that a high CR rate could be achieved for patients with R/R B-ALL treated with anti-CD19 CAR T-cell therapy, including patients with high-risk features. The relapse rate for subgroups with high-risk features after CAR T-cell treatment alone remains high, and CAR T-cell therapy followed by subsequent consolidative Allo-SCT has shown better LFS and OS in their study.

## 4. Relapse after CAR-T

Relapse after CARs therapy can be divided into two groups based on the flow cytometry assessment of CD19 expression on B-ALL: CD19-negative relapses and CD19-positive relapses [44,45]. CD19-positive relapses are often due to low potency and/or loss of CAR T cells. Several factors limit the power and efficacy of CAR-T cells, including the limited long-term persistence [46], the immune-suppressive tumor microenvironment [47], and intrinsic dysfunction associated with T-cell exhaustion [48,49].

While most relapses are CD19+, some ALL tumors evade CAR-T cell-mediated recognition and clearance by loss of expression of CD19 on the tumor cell surface. Sotillo et al. [50] looked at the genetic/epigenetic mechanisms of CD19- negative relapses by examining tumor samples from patients with CD19-negative disease. In these patients, the authors found deletions in CD19 locus and de novo frameshift and missense mutations in exon 2 of CD19. They also discovered lower levels of SRSF3 (a splicing factor whose function is to retain exon 2) in patients with R/R ALL, which allowed exon 2 skipping in tumors, producing a truncated CD19 variant that allowed tumor cells to escape killing by CAR-T cells. According to the authors, the underlying mechanism for relapse in these tumors was selecting preexisting alternatively spliced variants. Grupp et al. [28] described the phenomenon of “selection by immune pressure” in ALL patients treated with CAR-T cells.

## 5. Future Developments

### 5.1. CD19 CAR-T: Ongoing Trials

Numerous trials are underway with CARs (Table 4). OBERON (NCT03628053) trial aims to compare the benefits and risks of Tisacel to blinatumomab or inotuzumab in adult patients with R/R ALL [51]. This study is a phase III, open-label, multinational, randomized trial. Eligible patients will be randomized to the Tisacel treatment strategy (Tisacel after optional bridging chemotherapy and lymphodepleting chemotherapy) or control arm treatment (blinatumomab or inotuzumab per investigator’s discretion after optional bridging chemotherapy).

CASSIOPEIA (NCT03876769) is a single-arm, open-label, multi-center, phase II study to determine the efficacy and safety of Tisacel in de novo high-risk pediatric and young adult B-ALL patients who received first-line treatment and are MRD positive after first-line therapy [52]. 

Roddie et al. presented at 2020 ASH annual meeting results of 19 R/R B-ALL patients with a median age of 43 years (range 18–62), who received a novel CD19 CAR (CAT-41BBz CAR) (Phase I ALLCAR19-NCT02935257—a study of AUTO1) [53]. AUTO1 was a “fast-off” CAR-T cell therapy designed to minimize excessive activation and cytokine release, reducing toxicity. It also claims to reduce T cell exhaustion and enhance engraftment. The study showed a tolerable safety profile, despite the high disease burden of patients, and high remission rates with a CR rate of 84% with a negative MRD.

### 5.2. CD22 CAR T-Cell

Since CD19 negative relapses are seen in 10–20% of patients, CARs directed against other antigens, such as CD22, are being developed [54]. Pan et al. conducted a CD22 CAR T-cell therapy in 34 R/R B-ALL pediatric and adult patients who failed from previous CD19 CAR T-cell therapy [55]. The authors showed that treatment with a relatively low dosage of T-cells transduced with this CAR vector induced CR in 80% (24/30) patients that could be evaluated on day 30 (76% attained MRD-), and in 70.5% (24/34) of all enrolled patients. The authors observed low CRS and ICANS in most patients, self-limiting and needing no specific treatment, suggesting that CD22 CAR T-cell related toxicity was generally low. The authors concluded that CD22 CAR T-cells were capable of inducing a high remission rate in B-ALL patients who are R/R after chemotherapy, allo-SCT, and even CD19 CAR T-cell therapy. 

### 5.3. Dual-Target CARs

A strategy to overcome CD19-negative relapses is developing dual-target CARs by targeting CD19 and another antigen simultaneously, such as CD22 or CD20. 

The anti-CD20-CD19 bispecific CAR induced a full T-cell response upon engagement of CD19 or CD20 on target cells showing a real “OR” gate recognition in redirecting T-cell activation [56]. The bispecific CAR T cells cleared pediatric ALL with a mixed CD19+CD20+/CD20- phenotype from the blood and bone marrow of transplanted mice, while anti-CD20 CAR T cells left CD20- leukemic cells behind without curing the disease. Data indicate the superior anti-leukemic activity in the control of leukemia, implying that the anti-CD20-CD19 bispecific CAR T cells may reduce the risk of relapse through antigen-loss leukemic cells in the long term.

Dai et al. reported the design of a bispecific CAR simultaneous targeting of CD19 and CD22 [57]. The authors performed a phase 1 trial in R/R B-ALL and demonstrated bispecific CD19/CD22 CAR T cells could trigger robust cytolytic activity against target cells. CR with MRD-negative was achieved in 6 out of 6 enrolled patients. Autologous CD19/CD22 CAR T cells increased in vivo and were detected in the blood, bone marrow, and cerebrospinal fluid. No ICANS occurred in any of the six patients treated. One patient had a relapse with blast cells that no longer expressed CD19 and exhibited diminished CD22 site density approximately five months after treatment. The conclusions were that autologous CD19/CD22 CAR T cell therapy was feasible and safe and mediated potent anti-leukemic activity in patients with R/R B-ALL. Schultz and colleagues presented at the 2019 ASH annual meeting the results of a phase I clinical trials of CD19/CD22 bispecific CAR T cells in 19 patients (10 pediatric; 9 adults) with a median age of 23 years (range, 2–68) and median of 4 (range, 2–11) last lines for ALL [58]. Researchers utilized lentiviral transduction of a bivalent CAR construct incorporating the fmc63 CD19 and m971 CD22 single-chain variable fragments (scFvs) and a 41BB co-stimulatory endo domain. The authors tested two dose levels during dose escalation. Twelve patients have reached day 28 and are included in the safety and response analysis. 75% of them experienced CRS, and 17% developed ICANS. The CRS and ICANS were all grade 1 or 2 across both dose levels and pediatric and adult patients except for one adult with high disease burden who experienced grade 4 CRS and grade 4 ICANS, both of which were reversible. No differences in toxicities were seen across the patient age spectrum, and there were no treatment-related mortality cases within 28 days following CAR T infusion. Eleven of 12 (92%) patients achieved a CR, and OS for all infused patients was 92% with a median follow-up of 9.5 months from infusion time (range, 1–20). The authors concluded that the combined pediatric and adult phase I trials of bispecific CD19/CD22 targeting CAR T cells in R/R ALL demonstrates safety and tolerability at two dose levels. 

Yang et al. reported at the 2020 ASH Annual Meeting data on an anti-CD19/CD22 dual CAR-T (GC022F) therapy in patients with B-ALL based on a novel manufacturing platform, from a phase I clinical study in treating patients with B-ALL [59]. GC022F cells were manufactured using a novel FasTCAR^TM^ platform, which takes 24 h. In contrast, the conventional CD19/CD22 dual CAR-T (GC022C) cells used as parallel control in the preclinical study were manufactured by a traditional process that typically takes about two weeks. All patients received a conditioning regimen of fludarabine and cyclophosphamide before GC022F infusion. Compared with the GC022C, GC022F cells showed less exhaustion, younger phenotypes, higher expansion fold at in vitro culture, and high anti-leukemia efficacy in mice models. Comparing in vivo, GC022F treatment was more potent and could reduce tumor burden earlier and faster, leading to the significantly prolonged OS of the experimental animals. Nine children and one adult with B-ALL were enrolled and infused with GC022F. The median age was 10 (3–48) years, and the median bone marrow (BM) blasts were 21.0 (0.1–63.5)% at enrollment. Three patients had prior CD19 CAR-T cell therapy history, and one of whom had prior allo-SCT. GC022F exerted a superior safety profile with no observed grade ≥3 CRS and ICANSin all patients. After GC022F infusion, 6/6 patients achieved CR by day 28, 5/6 with minimal residual disease (MRD)-negative CR. This study demonstrated that anti-CD19/CD22 dual CAR T-cells could be successfully manufactured by FasTCAR^TM^ technology in 24 h, with younger and less exhausted phenotypes. Moreover, the Dual FasTCAR-T cells showed more potent efficacy in a xenograft mouse model than the conventional dual CAR-T cells. 

Other trials targeting dual antigens are currently underway [60], including preclinical data regarding a dual CD19 and CD123 targeting CAR [61]. CD123, the interleukin three receptor, is strongly associated with leukemic stem cells and is expressed in around 80% of B-ALL blasts [62]. Yan et al. first showed that the CD19-CD123 CCAR had impressive cytotoxic activity against leukemic cell lines and patient tumors, ablating a variety of CD19-positive and CD123- positive leukemic cell lines [57]. The cCAR targeted and responded to various primary leukemia samples and efficiently ablated CD19+ CD123+ B-ALL cells and CD123dim AML cells in these samples. CD19-CD123 cCAR ablates over >90% of double-positive primary B-ALL blasts in 24 h, indicating the compound CAR’s exceptional targeting capacity in eliminating tumor cells.

CAR-T therapy targeting three targets- CD19, CD20, and CD22, are also under development for ALL [63]. Fousek and colleagues designed two trivalent CAR T cell products with exodomains derived from single-chain variable fragments targeting CD19, CD20, and CD22. Each CAR contains the 4-1BB and T-cell receptor zeta chains. Donor T cells were engineered to express the CARs using a retroviral system. Using primary B-ALL cells, they observed that trivalent CAR T cells killed ALL cells more robustly than CD19 CAR T cells. In multiple models of CD19 escape in primary ALL, they showed that trivalent CAR T cells mitigated CD19 negative relapse, producing IFN-γ/TNF-α and killing CD19-negative primary ALL, while CD19 CAR T cells remained ineffective. 

A chondroitin sulfate proteoglycan 4 (CSPG4) membrane surface receptor has been found on mixed-lineage leukemia (MLL) rearranged B-ALL cells. A CSPG4-specific CAR is an active area of investigation for MLL rearranged B-ALL [64].

## 6. Allogeneic CAR-T

Allogeneic Cytokine Induced Killer (CIK) cells, a T-cell population characterized by the enrichment of CD3+CD56+ cells, have demonstrated a high safety profile in ALL patients. CIK cells could be skillfully engineered by the non-viral Sleeping Beauty (SB) transposon for the clinical application [65,66]. An Italian group generated CIK cells from 50 mL of donor-derived peripheral blood (PB) by electroporation with the GMP-grade CD19.CAR/pTMNDU3 and pCMV-SB11 plasmids [67]. After lymphodepletion with Fludarabine and Cyclophosphamide, four pediatric and seven adult patients withB-ALL patients relapsed after Allo-SCT received a single dose CARCIK-CD19 (*n* = 2 HLA identical sibling, *n* = 4 MUD, *n* = 5 haploidentical donor). Toxicities reported were a grade I CRS and an infusion-related DMSO-associated seizure, with the absence of dose-limiting toxicities, ICANS, and graft-versus-host disease (GvHD) in any of the treated patients. Four out of 5 patients receiving the highest doses achieved CR and CRi on day 28. The three patients in CR were also MRD-while the CRi was MRD+ and relapsed at day+49. The robust expansion was achieved in most patients as defined by detectable CAR T-cell detection in PB. The median time to peak engraftment was 14 days. The magnitude of expansion correlated with the CD19+ burden in the BM at the infusion time. CD8+ T cells represented the predominant CARCIK-CD19 T-cell subset (78.88% ± 5.33% D14 *n* = 6) along with CD3+CD56+ CIK cells and CD4+ T cells to a lesser extent. The majority of CAR T cells had a central and effector memory phenotype. CAR T cells were measurable up to 6 months with a mean persistence of 70.5 ± 14.85 days (follow up ranging from 28 days to 1 year). No significant difference was observed by integration analyses of the patients PB and cell products. 

The use of CAR-T therapies on a larger proportion of patients has been limited by variability of the final cell product, feasibility concerns, cost, and toxicity. Off-the-shelf allo products offer the opportunity to address some of these concerns. Allo products have their theoretical limitations, including the potential for graft-versus-host disease (GvHD) causing additional toxicity and host-versus-graft rejection limiting the efficacy.

Zhang et al. presented at the 2020 ASH Annual Meeting the CAR T-cells results derived from related donors in 37 patients (median age 19 years, range 3–61), with R/R B-ALL [68]. Of the 37 patients, 28 experienced a relapse following allo-SCT and whose lymphocytes were collected from their transplant donors (3 HLA matched sibling and 25 haploidentical). For the remaining nine patients without a prior transplant, the lymphocytes were collected from HLA identical sibling donors (*n* = 5) or haploidentical donors (*n* = 4) because CAR-T cells manufacture from patient samples either failed (*n* = 5) or blasts in peripheral blood were too high to collect quality T-cells. The authors showed that manufacturing CD19+ CAR-T cells derived from donors were feasible. For patients who relapse following allo-HSCT, the transplant donor-derived CAR-T cells were safe and effective with a CR rate as high as 96.4%. The authors concluded that the efficacy of CAR T-cells from haploidentical donors was inferior.

UCART22 is a genetically modified T-cell product manufactured from non-HLA matched healthy donor cells. Donor-derived T-cells are transduced using a lentiviral vector to express anti-CD22 CAR (anti-CD22 scFv-41BB-CD3ζ) and are further modified using Cellectis’ TALEN^®^ technology to disrupt the T-cell receptor alpha constant (TRAC) and CD52 genes. These modifications minimize graft versus host disease (GvHD) and allow anti-CD52 directed drugs in the lymphodepletion (LD). UCART22 demonstrated antigen-specific cytotoxic activity against B-ALL cell lines and primary human samples in vitro. BALLI-01 (NCT04150497) is a Phase I open-label dose-escalation study designed to assess the safety, the maximum tolerated dose (MTD), and preliminary anti-leukemic activity of UCART22 in patients with R/R B-ALL. Jain et al. published the preliminary results of 5 patients who received infusion [69]. UCART22 demonstrated no unexpected toxicities at tested doses. CRS was observed in 3 patients, all grade 1–2, and was manageable. No pts had DLT, GvHD, nor ICANS. Two pts achieved CRi. As host immune recovery was observed early, the addition of alemtuzumab to FC LD is now being explored in ongoing treatment cohorts to potentially achieve a more profound and more sustained T-cell depletion and promote expansion and persistence of UCART22. 

PBCAR0191 is a novel CD19 targeted allogeneic CAR T therapy candidate being developed for the treatment of R/R B-cell acute lymphoblastic leukemia (B-ALL) and non-Hodgkin lymphoma (NHL). PBCAR0191 was designed to limit the risk of GvHD by precisely inserting a CD19 specific CAR into the TRAC (T cell receptor alpha constant) locus in cells harvested from healthy donors. Those cells are then expanded. A CD3 elimination step is performed, followed by another expansion. Then, PBCAR0191 is vialed and frozen for shipment, then thawing, dilution, and infusion at the treatment site. Currently, it is in phase I/IIa clinical trial [70].

## 7. Allo-SCT after CAR-T

Although anti-CD19 CAR T-cell therapy shows promising efficacy in patients with R/R B-ALL, it fails to improve long-term DFS. Allo-SCT after CAR T-cell therapy has emerged as a promising strategy to prolong DFS. Nevertheless, which patients are likely to benefit from consolidative Allo-SCT remain to be studied. Real-life studies highlight that patients with poor risk disease were at increased risk of relapse after CAR T-cell therapy [71]. Elevated pre-lymphodepletion lactate dehydrogenase, low pre-lymphodepletion platelet count, absence of fludarabine in lymphodepletion, and persistent leukemic sequence high throughput sequencing in bone marrow after CAR T-cell treatment and early loss of CAR T cells have also been linked to relapse after CARs infusion. In patients having these risk factors, a consolidation with Allo-SCT after CAR T-cell therapy may prolong DFS. 

## 8. Expert Opinion

The cellular immunotherapy field was still in its infancy in 2012 when the first pediatric patient received the CAR-T therapy CTL019, now known as Tisacel. Since those years, trial activity has increased dramatically and continues at a rate of nearly 100 new trial registrations each year. There are no comparative studies between different anti-CD19 CAR T-cell constructs, and we can assess their efficacy and toxicity from meta-analyses and mechanistic studies. While Tisacel contains the 4-1BB co-stimulatory domain [22], MSKCC conducted a trial using a CAR construct with a CD28 co-stimulatory domain [29], and KTE-X19 is another CD19 CAR-T product with a CD28 co-stimulatory domain [39]. The 4-1BB co-stimulatory domain CAR-T shows more durable in vivo persistence than the CD28 co-stimulatory domain [49]. The trials varied widely by CAR vector constructs, manufacturing, eligibility criteria, patient population, and dosing schemes. Despite these differences, the studies have consistently shown that CD19 CAR-T therapy induces high CR rates in high-risk, heavily pretreated patients with R/R B-ALL. Although Kymriah (Tisacel) is the only approved therapy available in the ALL market, additional studies and comparison trials must further determine differences in efficacy between different anti-CD19 CAR T-cell constructs. Moreover, hematologists begin to have real-life data available, thanks to international registers. Real-life highlights how the patients treated have worse clinical characteristics than those included in studies. Despite this, the clinical outcomes of real-life demonstrate similar results to those of clinical trials. An exciting finding is that the rate of CRS and ICANS ≥ 3 is lower in real life than in clinical trials, so much to suggest a possible use of CAR-T therapy, at least in part, in outpatient setting. These results may be explained by the early use of tocilizumab or steroids. On the other hand, our difficulty in comparing toxic events between different constructs and real-life could be explained partly by the variability in the CRS grading criteria used in the various studies. Recently, Pennisi et al. [72] compared the five major CRS grading systems’ performance with the recently released American Society for Transplantation and Cellular Therapy CRS grading criteria. Agreement between the different grading systems was found only in 25% of patients, resulting in various toxicity management recommendations. The Penn grading system tended to upgrade mild CRS, and we can observe discrepancies between the MSKCC, CAR-T toxicity (CARTOX), and ASTCT criteria with regards to hypotension, and between the Lee, CARTOX, and ASTCT criteria with regards to organ damage [73,74,75,76]. We are still learning about what causes this toxicity, but inflammation plays definitely an important role [74]. Moreover, some patients experience delayed cytopenias, which is sometimes related to the degree of intensity of the lymphodepletion regimen that they were given. Moreover, normal B cells can be reduced with this approach, leading to B-cell aplasia, although this is only temporary. For CAR T-cell therapies to be feasible across institutions, clinicians’ comprehensive training, and a standardized approach to CRS management will be necessary to maximize safety and represent a priority for continued research. 

Other challenges need to be addressed. Future trials are needed to identify predictors of response, improve the risk-benefit balance, and minimize unnecessary financial outlay for individual patients and healthcare systems [77]. This topic could result in those predicted to respond poorly to chemotherapy but well to CAR-T cells receiving them upfront. Despite promising response rates in trials, applying this data to real-world patients is challenging, partly as inclusion criteria favor better prognosis groups. Comparative analysis of outcomes in patient cohorts with varying disease burden demonstrates an inferior CR, EFS, and OS in patients with high disease burden compared to patients with lower disease burden or no detectable disease at last evaluation before CAR infusion. Moreover, baseline platelet count, lactate dehydrogenase, and lymphodepletion regimen impacted EFS in MRD patients with negative CR after CD19 CAR T-cell.

With the longer follow-up and durable T cell persistence now reported, we are closer to answering the question of whether sustained remissions are possible with CAR T cell monotherapy. As might be expected, with a highly effective therapy using a single mechanism of action, escape pathways have emerged. Nevertheless, despite initial impressive in-depth responses obtained with this therapy, more than half of patients experienced relapse, with relapse rates as high as 65–85% in various studies. With these premises, it is our opinion that CAR-T can represent a bridge therapy to Allo-SCT in patients eligible for this procedure. Allo-SCT after CD19 CAR-T cell therapy is well tolerated and may improve EFS and provide optimal clinical benefit in patients with MRD-negative CR, typically within three months after CAR T-cell therapy. Different studies included variable numbers of patients that proceeded to Allo-SCT after anti-CD19 CAR T-cell infusion, ranging from 0% to 33%. Some protocols allowed Allo-SCT at the discretion of the treating clinician. Future trials and long-term outcome data are required to establish the role of Allo-SCT after CAR T-cell therapy.

Another question where we have no clear answer is whether CD19 must be the only target of CAR-T. CD19 is one target, but several other targets are being examined, such as CD20 and CD22. CD22-directed CAR-T cells have shown efficacy against leukemia as well in a recent clinical trial, representing the first alternative CAR target to approach comparable efficacy to CD19 CAR-T cells. CARs directed against other antigens may salvage some of these relapses and are in development for B-cell leukemias. Dual-targeting CARs or combination therapy may prevent relapses due to escape variants and, therefore, the way forward. If we only target one antigen, it is possible that leukemia can escape by no longer expressing that target. However, if we target two antigens, we can increase the likelihood that leukemia will not relapse. This method is still early in development, and combinatorial approaches are needed to anticipate and prevent this mode of relapse. 

What is the future of CAR T-cell therapy? Suppose anyone hazards a guess as to what the future will look like. In that case, off-the-shelf products seem to be the way to go because we can avoid apheresis and the waiting period to manufacture autologous CAR T cells. Even though this period can be as short as 2–3 weeks, this is a long time for patients with R/R disease. To transition to off the shelf products does have some nuances that need to be addressed. None of these products has obtained FDA approval yet, but several ongoing clinical trials investigate these approaches. A new “FasT” platform, which uses electroporation to transduce the CAR gene and has shortened the CAR-T cell manufacturing process by more than 24 h, has shown superior expansion capability and younger/less exhausted phenotypes a phase I clinical trial [78]. Other ways to mitigate this obstacle is to develop allogeneic “off the shelf” therapy [79]; however, allogeneic cells bear the risk of immune rejection by host T cells, as well as allo-reactivation of the CAR-T cells via the TCR receptor against host tissues, causing GVHD [80]. Many trials are currently enrolling “off the shelf” products, including a few trials with gene-edited deletion of the surface TRAC molecule to prevent GVHD [81].

The last issue is cost. Since the first CAR T-cell therapies gained FDA approval in 2017, the one-time treatments have led to unprecedented response rates in patients with B-cell ALL, with remarkable price tags of about $373,000 for a single infusion. The price does not account for manufacturing the product or managing potential long-term complications, and managing other therapy lines after relapse. So, we worry about whether health systems can afford to pay for these therapies. The pharmaceutic company introduced an outcomes-based pricing model for the ALL indication for Tisacel: if the treatment does not work, no one pays for it. That proposal is exciting with many problems, the least of which is that the definition of “not working” is not clear. 

## 9. Conclusions

In conclusion, CAR-T therapy is specifically developed for each patient and involves reprogramming the patient’s immune system cells, which we can use to target their cancer. It is a highly complex and potentially risky treatment, but it has been shown in trials to cure some patients, even those with quite advanced cancers, and were other available therapies have failed. Although the high initial response rate with CD19-CAR-T cells in B-ALL, relapses occur in a significant fraction of patients. Current strategies to improve CAR-T cell efficacy focus on improved CAR-T cells in vivo, multispecific CARs to overcome immune escape, and new CAR designs. We can use CAR-T cells for bridging to Allo-SCT in patients with R/R B-ALL since we currently cannot distinguish those CAR-T cell that will persist without further therapy from those that are likely to be short-lived. Future improvements in CAR-T cell constructs may allow longer-term remissions without additional Allo-SCT. Uncertainty about the real costs for administering these relatively new treatments is tied to questions about reimbursement.

## Figures and Tables

**Table 1:** CAR T-cell targets for the treatment of B acute lymphoblastic leukemia (ALL).

Antigen Target	CAR Structure
CD19	CD3ζ and CD28 or CD3ζ and 4-1BB
CD22	CD3ζ and CD28
CD20	CD3ζ or and CD3ζ and 4-1BB

**Table 2:** Clinical efficacy of CD19 CAR T-cells in relapsed/refractory B acute lymphoblastic leukemia in the main clinical trials.

Author (Years)	Population	T-Cell Product	Age, Med (Range)	NO.	Priorhsct	CR	Post-Carhsct	Survival
Park (2018)	Adults	19–28z	44 (23–74)	53	36%	83%	39%	39% relapse (24% CD19-) Median OS/EFS: 12.9/6.1 months
Hay (2019)	Adults	19–41BBz	39 (20–76)	53	43%	85%	40%	49% relapse (27% CD19-)
Buechner (2017)	Pediatrics	19–41BBz	12 (3–23)	68	61%	83%	12%	36% relapse (68% CD19-) 6 months EFS: 73%
Lee (2015)	Pediatrics	19–28z	14 (5–27)	20	38%	70%	71%	10 months OS: 51.6%
Gardner (2017)	Pediatrics	19–41BBz	12 (1–25)	45	62%	89%	24%	12 months EFS: 51% (45% relapse) 12 months OS: 70%

**Table 3:** Key anti-CD19 CAR T-cell therapy trials in relapsed/refractory B acute lymphoblastic leukemia.

	ELIANA	MSKCC	ZUMA-3
CAR T-cell agent	Tisagenleucel	JCAR015	Brexucabtagene autoleucel
Author (Years)	Maude (2018)	Park (2018)	Shah (2019)
Study phase	II	I	I/II
Study population	Pediatric/young adults	Adults	Adults
No. Patients	75	53	45
CR %	MRD negative: 81	Overall: 83	Overall: 68 RP2D: 84
Median OS, mos	19.1	12.9	--
Median EFS, mos	NR	6.1	--
Median DoR, mos	NR	--	RP2D: 12.9
Median follow-up, mos	13.1	29	16

DoR, duration of response; EFS, event-free survival; MRD, measurable residual disease; NR, not reached; RP2D, recommended phase II dose; mos, months.

**Table 4:** Select ongoing trials with CAR T-cell therapy for patients with ALL relapsed/refractory B acute lymphoblastic leukemia.

Trial	Phase	Treatment	Population	Endpoints
OBERON (NCT03628053)	III	Tisagenlecleucel vs. blinatumomab or inotuzumabozogamicin	Adults with B-cell precursor ALL; R/R after 1–2 lines of therapy or ASCT	OS
CASSIOPEIA (NCT03876769)	II	Tisagenlecleucel	Pediatric/young adult high-risk B-cell ALL; MRD positive after first-line therapy	DFS
ZUMA-4 (NCT02625480)	I/II	Axicabtageneciloleucel	Pediatric/adolescent pts with R/R B-precursor ALL or R/R B-cell NHL	AEs, CRR, ORR

## Data Availability

Not applicable.

## References

[1] Ward E., DeSantis C., Robbins A., Kohler B., Jemal A. (2014). Childhood and adolescent cancer statistics, 2014. CA Cancer J. Clin..

[2] Cancer Stat Facts: Leukemia—Acute Lymphocytic Leukemia (ALL). https://seer.cancer.gov/statfacts/html/alyl.html.

[3] Bennett J.M., Catovsky D., Daniel M.T., Flandrin G., Galton D.A., Gralnick H.R., Sultan C. (1976). Proposals for the classification of the acute leukemias. French-American-British (FAB) co-operative group. Br. J. Haematol..

[4] Harris N.L., Jaffe E.S., Diebold J., Flandrin G., Muller-Hermelink H.K., Vardiman J., Lister T.A., Bloomfield C.D. (1999). World Health Organization classification of neoplastic diseases of the hematopoietic and lymphoid tissues: Report of the Clinical Advisory Committee meeting-Airlie House, Virginia, November 1997. J. Clin. Oncol..

[5] Vardiman J.W., Thiele J., Arber D.A., Brunning R.D., Borowitz M.J., Porwit A., Harris N.L., Le Beau M.M., Hellström-Lindberg E., Tefferi A. (2009). The 2008 revision of the World Health Organization (WHO) classification of myeloid neoplasms and acute leukemia: Rationale and important changes. Blood.

[6] Arber D.A., Orazi A., Hasserjian R., Thiele J., Borowitz M.J., Le Beau M.M., Bloomfield C.D., Cazzola M., Vardiman J.W. (2016). The 2016 revision to the World Health Organization classification of myeloid neoplasms and acute leukemia. Blood.

[7] Terwilliger T., Abdul-Hay M. (2017). Acute lymphoblastic leukemia: A comprehensive review and 2017 update. Blood Cancer J..

[8] Rowe J.M., Buck G., Burnett A.K., Chopra R., Wiernik P.H., Richards S.M., Lazarus H.M., Franklin I.M., Litzow M.R., Ciobanu N. (2005). Induction therapy for adults with acute lymphoblastic leukemia: Results of more than 1500 patients from the international ALL trial: MRC UKALL XII/ECOG E2993. Blood.

[9] Kantarjian H., Thomas D., O’Brien S., Cortes J., Giles F., Jeha S., Bueso-Ramos C.E., Pierce S., Shan J., Koller C. (2004). Long-term follow-up results of hyperfractionated cyclophosphamide, vincristine, doxorubicin, and dexamethasone (Hyper-CVAD), a dose-intensive regimen, in adult acute lymphocytic leukemia. Cancer.

[10] Linker C., Damon L., Ries C., Navarro W. (2002). Intensified and shortened cyclical chemotherapy for adult acute lymphoblastic leukemia. J. Clin. Oncol..

[11] Fielding A.K., Richards S.M., Chopra R., Lazarus H.M., Litzow M.R., Buck G., Durrant I.J., Luger S.M., Marks D.I., Franklin I.M. (2007). Medical Research Council of the United Kingdom Adult ALL Working Party; Eastern Cooperative Oncology Group. The outcome of 609 adults after relapse of acute lymphoblastic leukemia (ALL); an MRC UKALL12/ECOG 2993 study. Blood.

[12] Tavernier E., Boiron J.M., Huguet F., Bradstock K., Vey N., Kovacsovics T., Delannoy A., Fegueux N., Fenaux P., Stamatoullas A. (2007). Outcome of treatment after first relapse in adults with acute lymphoblastic leukemia initially treated by the LALA-94 trial. Leukemia.

[13] Kantarjian H.M., De Angelo D.J., Stelljes M., Martinelli G., Liedtke M., Stock W., Gökbuget N., O’Brien S., Wang K., Wang T. (2016). Inotuzumab ozogamicin versus standard therapy for acute lymphoblastic leukemia. N. Engl. J. Med..

[14] Kantarjian H., Stein A., Gökbuget N., Fielding A.K., Schuh A.C., Ribera J.M., Wei A., Dombret H., Foà R., Bassan R. (2017). Blinatumomab versus chemotherapy for advanced acute lymphoblastic leukemia. N. Engl. J. Med..

[15] Ribrag V., Koscielny S., Bosq J., Leguay T., Casasnovas O., Fornecker L.M., Recher C., Ghesquieres H., Morschhauser F., Girault S. (2016). Rituximab and dose-dense chemotherapy for adults with burkitt’s lymphoma: A randomised, controlled, open-label, phase 3 trial. Lancet.

[16] Dai H., Wang Y., Lu X., Han W. (2016). Chimeric Antigen Receptors Modified T-Cells for Cancer Therapy. Natl. Cancer Inst..

[17] Jackson H.J., Rafiq S., Brentjens R.J. (2016). Driving CAR T-cells forward. Nat. Rev. Clin. Oncol..

[18] Kochenderfer J.N., Rosenberg S.A. (2013). Treating B-cell cancer with T cells expressing anti-CD19 chimeric antigen receptors. Nat. Rev. Clin. Oncol..

[19] Imai C., Mihara K., Andreansky M., Nicholson I.C., Pui C.H., Geiger T.L., Campana D. (2004). Chimeric receptors with 4–1BB signaling capacity provoke potent cytotoxicity against acute lymphoblastic leukemia. Leukemia.

[20] Maher J., Brentjens R.J., Gunset G., Rivière I., Sadelain M. (2002). Human T-lymphocyte cytotoxicity and proliferation directed by a single chimeric TCR zeta/CD28 receptor. Nat. Biotechnol..

[21] Savoldo B., Ramos C.A., Liu E., Mims M.P., Keating M.J., Carrum G., Kamble R.T., Bollard C.M., Gee A.P., Mei Z. (2011). CD28 costimulation improves expansion and persistence of chimeric antigen receptor-modified T cells in lymphoma patients. J. Clin. Investig..

[22] Maude S.L., Teachey D.T., Porter D.L., Grupp S.A. (2015). CD19-targeted chimeric antigen receptor T-cell therapy for acute lymphoblastic leukemia. Blood.

[23] Tedder T.F., Zhou L.J., Engel P. (1994). The CD19/CD21 signal transduction complex of B lymphocytes. Immunol. Today.

[24] Matsuo Y., Drexler H.G. (1998). Establishment and characterization of human B cell precursor-leukemia cell lines. Leuk. Res..

[25] Eshhar Z., Waks T., Gross G., Schindler D.G. (1993). Specific activation and targeting of cytotoxic lymphocytes through chimeric single chains consisting of antibody-binding domains and the gamma or zeta subunits of the immunoglobulin and T-cell receptors. Proc. Natl. Acad. Sci. USA.

[26] Van der Stegen S.J., Hamieh M., Sadelain M. (2015). The pharmacology of second-generation chimeric antigen receptors. Nat. Rev. Drug Discov..

[27] Vairy S., Garcia J.L., Teira P., Bittencourt H. (2018). CTL019 (tisagenlecleucel): CAR-T therapy for relapsed and refractory B-cell acute lymphoblastic leukemia. Drug Des. Devel. Ther..

[28] Grupp S.A., Kalos M., Barrett D., Aplenc R., Porter D.L., Rheingold S.R., Teachey D.T., Chew A., Hauck B., Wright J.F. (2013). Chimeric antigen receptor-modified T cells for acute lymphoid leukemia. N. Engl. J. Med..

[29] Park J.H., Rivière I., Gonen M., Wang X., Sénéchal B., Curran K.J., Sauter C., Wang Y., Santomasso B., Mead E. (2018). Long-Term Follow-up of CD19 CAR Therapy in Acute Lymphoblastic Leukemia. N. Engl. J. Med..

[30] Turtle C.J., Hanafi L.A., Berger C., Gooley T.A., Cherian S., Hudecek M., Sommermeyer D., Melville K., Pender B., Budiarto T.M. (2016). CD19 CAR-T cells of defined CD41:CD81 composition in adult B cell ALL patients. J. Clin. Investig..

[31] Hay K.A., Gauthier J., Hirayama A.V., Voutsinas J.M., Wu Q., Li D., Gooley T.A., Cherian S., Chen X., Pender B.S. (2019). Factors associated with durable EFS in adult B-cell ALL patients achieving MRD-negative CR after CD19 CAR T-cell therapy. Blood.

[32] Fry T.J., Shah N.N., Orentas R.J., Stetler-Stevenson M., Yuan C.M., Ramakrishna S., Wolters P., Martin S., Delbrook C., Yates B. (2018). CD22- targeted CAR T cells induce remission in B-ALL that is naive or resistant to CD19- targeted CAR immunotherapy. Nat. Med..

[33] Lee D.W., Kochenderfer J.N., Stetler-Stevenson M., Cui Y.K., Delbrook C., Feldman S.A., Fry T.J., Orentas R., Sabatino M., Shah N.N. (2015). T cells expressing CD19 chimeric antigen receptors for acute lymphoblastic leukaemia in children and young adults: A phase 1 dose-escalation trial. Lancet.

[34] Gardner R.A., Finney O., Annesley C., Brakke H., Summers C., Leger K., Bleakley M., Brown C., Mgebroff S., Kelly-Spratt K.S. (2017). Intent-to-treat leukemia remission by CD19 CAR T cells of defined formulation and dose in children and young adults. Blood.

[35] Maude S.L., Teachey D.T., Rheingold S.R., Shaw P.A., Aplenc R., Barrett D.M., Barker C.S., Callahan C., Frey N.V., Nazimuddin F. (2016). Sustained remissions with CD19- specific chimeric antigen receptor (CAR)- modified T cells in children with relapsed/refractory ALL. J. Clin. Oncol..

[36] Grupp S.A., Maude S.L., Shaw P.A., Aplenc R., Barrett D.M., Callahan C., Lacey S.F., Levine B.L., Melenhorst J.J., Motley L. (2015). Durable remissions in children with re- lapsed/refractory ALL treated with T cells engineered with a CD19-targeted chimeric antigen receptor (CTL019). Blood.

[37] Maude S.L., Laetsch T.W., Buechner J., Rives S., Boyer M., Bittencourt H., Bader P., Verneris M.R., Stefanski H.E., Myers G.D. (2018). Tisagenlecleucel in Children and Young Adults with B-Cell Lymphoblastic Leukemia. N. Engl. J. Med..

[38] Grupp S.A., Maude S.L., Rives S., Baruchel A., Boyer M.W., Bittencourt H., Bader P., Büchner J., Laetsch T.W., Stefanski H. (2018). Updated Analysis of the Efficacy and Safety of Tisagenlecleucel in Pediatric and Young Adult Patients with Relapsed/Refractory (r/r) Acute Lymphoblastic Leukemia. Blood.

[39] Shah B.D., Bishop M.R., Oluwole O.O., Logan A., Baer M.R., Donnellan W.B., Carr-O’Dwyer K.M., Holmes H., Arellano M.L., Ghobadi A. (2019). End of phase I results of ZUMA-3, a phase 1/2 study of KTE-X19, anti-CD19 chimeric antigen receptor (CAR) T cell therapy, in adult patients (pts) with relapsed/refractory (R/R) acute lymphoblastic leukemia (ALL). J. Clin. Oncol..

[40] Schultz L.M., Baggott C., Prabhu S., Pacenta H., Phillips C.L., Rossoff J., Stefanski H., Talano J.A., Moskop A., Margossian S.P. (2020). Disease Burden Impacts Outcomes in Pediatric and Young Adult B-Cell Acute Lymphoblastic Leukemia after Commercial Tisagenlecleucel: Results from Pediatric Real World CAR Consortium (PRWCC). Blood.

[41] Pasquini M.C., Hu Z.H., Curran K., Laetsch T., Locke F., Rouce R., Pulsipher M.A., Phillips C.L., Keating A., Frigault M.J. (2020). Real-world evidence of tisagenlecleucel for pediatric acute lymphoblastic leukemia and non-Hodgkin lymphoma. Blood Adv..

[42] Anagnostou T., Riaz I.B., Hashmi S.K., Murad M.H., Kenderian S.S. (2020). Anti-CD19 chimeric antigen receptor T-cell therapy in acute lymphocytic leukaemia: A systematic review and meta-analysis. Lancet Haematol..

[43] Zhang X., Lu X.A., Yang J., Zhang G., Li J., Song L., Su Y., Shi Y., Zhang M., He J. (2020). Efficacy and safety of anti-CD19 CAR T-cell therapy in 110 patients with B-cell acute lymphoblastic leukemia with high-risk features. Blood Adv..

[44] Gardner R., Wu D., Cherian S., Fang M., Hanafi L.A., Finney O., Smithers H., Jensen M.C., Riddell S.R., Maloney D.G. (2016). Acquisition of a CD19-negative myeloid phenotype allows immune escape of MLL- rearranged B-ALL from CD19 CAR-T-cell therapy. Blood.

[45] Park J.H., Riviere I., Wang X., Bernal Y.B., Yoo S., Purdon T., Halton E., Quintanilla H., Curran K.J., Sauter C.S. (2014). CD19-Targeted 19–28z CAR-modified autologous T cells induce high rates of complete remission and durable responses in adult patients with relapsed, refractory B-cell ALL. Blood.

[46] Fraietta J.A., Lacey S.F., Orlando E.J., Pruteanu-Malinici I., Gohil M., Lundh S., Boesteanu A.C., Wang Y., O’Connor R.S., Hwang W.T. (2018). Determinants of response and resistance to CD19 chimeric antigen receptor (CAR) T cell therapy of chronic lymphocytic leukemia. Nat. Med..

[47] Long A.H., Highfill S.L., Cui Y., Smith J.P., Walker A.J., Ramakrishna S., El-Etriby R., Galli S., Tsokos M.G., Orentas R.J. (2016). Reduction of MDSCs with all-trans retinoic acid improves CAR therapy efficacy for sarcomas. Cancer Immunol. Res..

[48] Lynn R.C., Weber E.W., Sotillo E., Gennert D., Xu P., Good Z., Anbunathan H., Lattin J., Jones R., Tieu V. (2019). c-Jun overexpression in CAR T cells induces exhaustion resistance. Nature.

[49] Long A.H., Haso W.M., Shern J.F., Wanhainen K.M., Murgai M., Ingaramo M., Smith J.P., Walker A.J., Kohler M.E., Venkateshwara V.R. (2015). 4–1BB costimulation ameliorates T cell exhaustion induced by tonic signaling of chimeric antigen receptors. Nat. Med..

[50] Sotillo E., Barrett D.M., Black K.L., Bagashev A., Oldridge D., Wu G., Sussman R., Lanauze C., Ruella M., Gazzara M.R. (2015). Convergence of acquired mutations and alternative splicing of CD19 enables resistance to CART-19 immunotherapy. Cancer Discov..

[51] Tisagenlecleucel Versus Blinatumomab or Inotuzumab for Adult Patients with Relapsed/Refractory B-Cell Precursor Acute Lymphoblastic Leukemia: A Randomized Open Label, Multicenter, Phase III Trial. https://clinicaltrials.gov/ct2/show/NCT03628053.

[52] A Phase II Trial of Tisagenlecleucel in First-Line High-Risk (HR) Pediatric and Young Adult Patients With B-Cell Acute Lymphoblastic Leukemia (B-ALL) Who Are Minimal Residual Disease (MRD) Positive at the End of Consolidation (EOC) Therapy. https://www.clinicaltrials.gov/ct2/show/NCT03876769.

[53] Roddie C., O’Reilly M.A., Marzolini M.A.V., Wood L., Dias J., Cadinanos Garai A., Bosshard L., Abbasian M., Lowdell M.W., Wheeler G. ALLCAR19: Updated Data Using AUTO1, a Novel Fast-Off Rate CD19 CAR in Relapsed/Refractory B-Cell Acute Lymphoblastic Leukaemia and Other B-Cell Malignancies. Proceedings of the 62nd ASH Annual Meeting and Exposition.

[54] Haso W., Lee D.W., Shah N.N., Stetler-Stevenson M., Yuan C.M., Pastan I.H., Dimitrov D.S., Morgan R.A., FitzGerald D.J., Barrett D.M. (2013). Anti-CD22-chimeric antigen receptors targeting B-cell precursor acute lymphoblastic leukemia. Blood.

[55] Pan J., Niu Q., Deng B., Liu S., Wu T., Gao Z., Liu Z., Zhang Y., Qu X., Zhang Y. (2019). CD22 CAR T-cell therapy in refractory or relapsed B acute lymphoblastic leukemia. Leukemia.

[56] Martyniszyn A., Krahl A.C., André M.C., Hombach A.A., Abken H. (2017). CD20-CD19 Bispecific CAR T Cells for the Treatment of B-Cell Malignancies. Hum. Gene. Ther..

[57] Dai H., Wu Z., Jia H., Tong C., Guo Y., Ti D., Han X., Liu Y., Zhang W., Wang C. (2020). Bispecific CAR-T cells targeting both CD19 and CD22 for therapy of adults with relapsed or refractory B cell acute lymphoblastic leukemia. J. Hematol. Oncol..

[58] Schultz L.M., Muffly L.S., Spiegel J.Y., Ramakrishna S., Hossain N., Baggott C., Sahaf B., Patel S., Craig J., Yoon J. Phase I Trial Using CD19/CD22 Bispecific CAR T Cells in Pediatric and Adult Acute Lymphoblastic Leukemia (ALL). Proceedings of the 61nd ASH Annual Meeting and Exposition.

[59] Yang J., Jiang P., Zhang X., Li J., Wu Y., Xu L., Su Y., Hu X., Zhao X., Dong Q. Successful 24-Hours Manufacture of Anti-CD19/CD22 Dual Chimeric Antigen Receptor (CAR) T Cell Therapy for B-Cell Acute Lymphoblastic Leukemia (B-ALL) Clinically Relevant Abstract. Proceedings of the 62nd ASH Annual Meeting and Exposition.

[60] Shah N., Maatman T., Hari P.N., Johnson B. (2019). Multi-targeted CAR- T cell therapies for B-cell malignancies. Front. Oncol..

[61] Yan L.E., Zhang H., Wada M., Fang L., Feng J., Zhang W., Chen Q., Cao Y., Pinz K.G., Chen K.H. (2020). Targeting Two Antigens Associated with B-ALL with CD19-CD123 Compound Car T Cell Therapy. Stem. Cell. Rev. Rep..

[62] Hassanein N.M., Alcancia F., Perkinson K.R., Buckley P.J., Lagoo A.S. (2009). Distinct expression patterns of CD123 and CD34 on normal bone marrow B-cell precursors (“hematogones”) and B lymphoblastic leukemia blasts. Am. J. Clin. Pathol..

[63] Fousek K., Watanabe J., George A., An X., Samaha H.S., Navai S.A., Byrd T.T., Jang A., Kim H., Sujith J. (2018). Targeting CD19-negative relapsed B-acute lymphoblastic leukemia using trivalent CAR T cells. J. Clin. Oncol..

[64] Harrer D.C., Schuler G., Dörrie J., Schaft N. (2019). CSPG4-specific CAR T cells for high-risk childhood B cell precursor leukemia. Int. J. Mol. Sci..

[65] Magnani C.F., Mezzanotte C., Cappuzzello C., Bardini M., Tettamanti S., Fazio G., Cooper L.J.N., Dastoli G., Cazzaniga G., Biondi A. (2018). Preclinical Efficacy and Safety of CD19CAR Cytokine-Induced Killer Cells Transfected with Sleeping Beauty Transposon for the Treatment of Acute Lymphoblastic Leukemia. Hum. Gene. Ther..

[66] Biondi A., Magnani C.F., Tettamanti S., Gaipa G., Biagi E. (2017). Redirecting T cells with Chimeric Antigen Receptor (CAR) for the treatment of childhood acute lymphoblastic leukemia. J. Autoimmun..

[67] Magnani C.F., Gaipa G., Lussana F., Belotti D., Gritti G., Napolitano S., Buracchi C., Borleri G.M., Zaninelli S., Rizzuto G. Donor-Derived CAR T Cells Engineered with Sleeping Beauty Achieve Anti-Leukemic Activity without Severe Toxicity. Proceedings of the 62nd ASH Annual Meeting and Exposition.

[68] Zhang X., Yang J., Li W., Zhang G., Su Y., Shi Y., Song D., Zhang M., He J., Xu L. (2020). Feasibility, and Efficacy of Donor-Derived cd19-Targeted Car t-Cell Therapy in Refractory/Relapsed(r/r)b-Cell Acute Lymphoblastic Leukemia (b-all) Patients. Blood.

[69] Jain N., Roboz G.J., Konopleva M., Liu H., Jabbour E., Poirot C., Schiffer-Manniou C., Gouble A., Haider A., Zernovak O. (2020). Preliminary Results of Balli-01: A Phase I Study of UCART22 (allogeneic engineered T-cells expressing anti-CD22 Chimeric Antigen Receptor) in Adult Patients with Relapsed or Refractory (R/R) CD22+ B-Cell Acute Lymphoblastic Leukemia (B-ALL). Blood.

[70] Jacobson C.A., Herrera A.F., Budde L.E., DeAngelo D.J., Heery C., Stein A., Jain M.D., Bijal Shah B. (2019). Initial Findings of the Phase 1 Trial of PBCAR0191, a CD19 Targeted Allogeneic CAR-T Cell Therapy. Blood.

[71] Jiang H., Hu Y., Mei H. (2020). Consolidative allogeneic hematopoietic stem cell transplantation after chimeric antigen receptor T-cell therapy for relapsed/refractory B-cell acute lymphoblastic leukemia: Who? When? Why?. Biomark. Res..

[72] Pennisi M., Jain T., Santomasso B.D., Mead E., Wudhikarn K., Silverberg M.L., Batlevi Y., Shouval R., Devlin S.M., Batlevi C. (2020). Comparing CAR T-cell toxicity grading systems: Application of the ASTCT grading system and implications for management. Blood Adv..

[73] Maude S.L., Barrett D., Teachey D.T., Grupp S.A. (2014). Managing cytokine release syndrome associated with novel T cell-engaging therapies. Cancer J..

[74] Neelapu S.S., Tummala S., Kebriaei P., Wierda W., Gutierrez C., Locke F.L., Komanduri K.V., Lin Y., Jain N., Daver N. (2018). Chimeric antigen receptor T-cell therapy— assessment and management of toxicities. Nat. Rev. Clin. Oncol..

[75] Mahadeo K.M., Khazal S.J., Abdel-Azim H., Fitzgerald J.C., Taraseviciute A., Bollard C.M., Tewari P., Duncan C., Traube C., McCall D. (2019). Management guidelines for paediatric patients receiving chimeric antigen receptor T cell therapy. Nat. Rev. Clin. Oncol..

[76] Lee D.W., Santomasso B.D., Locke F.L., Ghobadi A., Turtle C.J., Brudno J.N., Maus M.V., Park J.H., Mead E., Pavletic S. (2019). ASTCT consensus grading for cytokine release syndrome and neurologic toxicity associated with immune effector cells. Biol. Blood Marrow Transplant..

[77] Du M., Hari P., Hu Y., Mei H. (2020). Biomarkers in individualized management of chimeric antigen receptor T cell therapy. Biomark. Res..

[78] Yang J., He J., Zhang X., Wang Z., Zhang Y., Cai S., Sun Z., Ye X., He Y., Shen L. (2019). A Feasibility and Safety Study of a New CD19-Directed Fast CAR-T Therapy for Refractory and Relapsed B Cell Acute Lymphoblastic Leukemia. Blood.

[79] Benjamin R., Graham C., Yallop D., Jozwik A., Ciocarlie O., NJain N., Jabbour E.J., Maus M.V., Frigault M., Boissel N. (2018). Preliminary Data on Safety, Cellular Kinetics and Anti-Leukemic Activity of UCART19, an Allogeneic Anti-CD19 CAR T-Cell Product, in a Pool of Adult and Pediatric Patients with High-Risk CD19+ Relapsed/Refractory B-Cell Acute Lymphoblastic Leukemia. Blood.

[80] Qasim W. (2019). Allogeneic CAR T cell therapies for leukemia. Am. J. Hematol..

[81] Poirot L., Philip B., Schiffer-Mannioui C., Le Clerre D., Chion-Sotinel I., Derniame S., Potrel P., Bas C., Lemaire L., Galetto R. (2015). Multiplex genome-edited T-cell manufacturing platform for “off-the-shelf” adoptive T-cell immunotherapies. Cancer Res..

